# Current Regulatory Frameworks for Bioactive Inputs in Plant Systems Producing Pharmaceutically Important Compounds

**DOI:** 10.3390/plants15142204

**Published:** 2026-07-19

**Authors:** Aldo Cordoba, Karen Esquivel, Ana Angélica Feregrino-Pérez

**Affiliations:** 1 Graduate and Research Division, Faculty of Engineering, Universidad Autónoma de Querétaro, Cerro de las Campanas, Santiago de Querétaro 76017, Qro., Mexico; acordoba07@alumnos.uaq.mx; 2 Department of Quantitative Methods, Universidad Anahuac Campus Querétaro, El Marqués 76246, Qro., Mexico

**Keywords:** elicitors, plant biostimulants, bioinputs, biofertilizers, regulatory frameworks, specialized metabolites

## Abstract

Plant cell, tissue, and organ cultures are increasingly used as controlled platforms for producing pharmaceutically important specialized metabolites. Their productivity can be improved through elicitors, plant biostimulant-like substances, microbial inputs, and nano-enabled delivery systems. However, the regulatory interpretation of these inputs is not determined only by their biological mechanism. It also depends on product composition, formulation, intended use, exposure scenario, and label claim. This review analyzes how elicitors, plant biostimulants, biofertilizer- and bioinput-related concepts, and nano-enabled inputs are positioned within selected regulatory frameworks in the European Union, United States, Canada, Brazil, India, China, and Mexico. The analysis shows that current frameworks increasingly emphasize product identity, efficacy substantiation, safety evidence, and labeling, but they remain fragmented for multifunctional materials that may simultaneously affect nutrient-related processes, abiotic stress tolerance, defense signaling, growth regulation, and specialized metabolism. Nano-enabled and encapsulated formulations create additional challenges because they can modify uptake, persistence, bioavailability, release behavior, residue profiles, and non-target exposure compared with conventional formulations. A clearer distinction is therefore needed between contained biotechnological use in plant biofactories and open-environment agricultural applications. Future regulatory development should move toward formulation-level assessment, claim-specific evidence, nanospecific characterization, traceability, and harmonized terminology to support innovation while ensuring safety, reproducibility, and regulatory clarity.

## 1. Introduction: Plant Cell Biofactories and the Regulatory Challenge

Plant-derived specialized metabolites constitute one of the most chemically diverse reservoirs of bioactive molecules for pharmaceutical, nutraceutical, cosmetic, and agricultural applications [1,2]. Alkaloids, terpenoids, phenolics, glycosides, saponins, and other specialized metabolites often exhibit complex stereochemistry and biological activities that are difficult to reproduce through conventional chemical synthesis or alternative production platforms [3]. Representative examples include taxanes from *Taxus* spp. [4], vinca alkaloids from *Catharanthus roseus* [5], artemisinin from *Artemisia annua* [6], ginsenosides from *Panax* spp. [7], and shikonin derivatives from *Lithospermum erythrorhizon* [8], all of which illustrate the pharmacological value and structural diversity of plant-derived compounds. However, their natural accumulation in plants is frequently low, tissue-specific, and strongly influenced by genotype, geography, seasonality, developmental stage, biotic interactions, and abiotic stress [9]. These limitations have historically constrained the reliable supply of high-value compounds from wild or field-grown medicinal plants, while also raising concerns related to overharvesting, environmental variability, and batch-to-batch inconsistency [3]. In this context, plant cell, tissue, and organ culture systems have emerged as controlled and renewable platforms for the production of specialized metabolites independent of environmental and geographical constraints [10,11].

Plant cell biofactories include callus cultures, cell suspension cultures, hairy root cultures, adventitious root cultures, and related in vitro systems that can be scaled in bioreactors under sterile and highly controlled conditions. Compared with whole-plant cultivation, these platforms allow tighter control over nutrient composition, phytohormone balance, light conditions, oxygen transfer, pH, biomass accumulation, and harvest timing [12]. Recent advances have further expanded the potential of plant cell, tissue, and organ culture systems by integrating metabolomics, computational modelling, and artificial intelligence to monitor biosynthetic responses and support the transition from empirical optimization toward more rational process design [11,13].

Commercial and semi-commercial examples have demonstrated the feasibility of these systems. *Taxus* cell cultures have been developed for taxane and paclitaxel production [4], while *Lithospermum erythrorhizon* cell cultures represent one of the earliest examples of industrial production of a plant specialized metabolite, shikonin, by dedifferentiated cell cultures [14]. Similarly, *Panax*-derived cultures have been explored for ginsenoside accumulation, illustrating the relevance of in vitro platforms for high-value triterpenoid saponins [7]. Nevertheless, broader industrial adoption remains constrained by productivity, long-term culture stability, scale-up performance, downstream processing, and regulatory uncertainty [15,16].

The productivity of plant biofactories can be improved through complementary strategies such as cell line selection, precursor feeding, culture optimization, and elicitation [17,18]. Among these approaches, elicitation has received particular attention because many pharmaceutically relevant specialized metabolites are associated with plant defense [19], stress adaptation [20], and health benefits [21]. Biotic and abiotic elicitors, including jasmonates, salicylates [22], chitosan derivatives [23], oligosaccharides [24], cyclodextrins, microbial signals, metallic ions, physical stimuli, and nanoparticles, can activate defense-related signaling cascades and redirect carbon flux toward specific metabolite classes [25].

However, elicitor responses are highly dependent on chemical identity, concentration, exposure time, developmental stage, cell line, culture system, and downstream extraction strategy [26,27]. Therefore, elicitation should not be regarded merely as an additive treatment, but as a process-defining variable capable of influencing biological activity, productivity, reproducibility, product composition, and, in some cases, regulatory classification.

**Figure 1:**
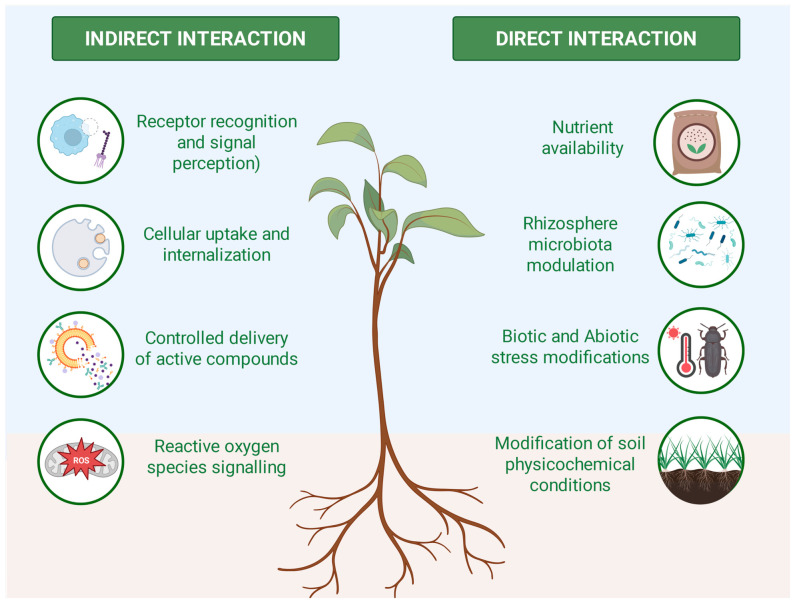
Direct and indirect mechanisms by which bioactive inputs interact with plant systems. Created in BioRender (https://www.biorender.com/).

Bioactive inputs like elicitors may act on plant systems through both direct and indirect mechanisms [28]. Figure 1 summarizes the main direct and indirect interaction pathways through which elicitors, biostimulant-like compounds, microbial inputs, and nano-enabled formulations can influence plant performance and specialized metabolism. Direct effects may include perception by plant receptors, modulation of reactive oxygen species, hormonal crosstalk, activation of defense-related transcription factors, and induction of biosynthetic genes [29]. Indirect effects may involve changes in nutrient availability, rhizosphere interactions, microbial signaling, stress priming, or improved delivery and persistence of the active compound [30]. For instance, chitosan may act as a defense elicitor and stress modulator, microbial inputs may alter nutrient cycling or produce signaling metabolites, and nanoscale carriers may modify uptake, release kinetics, and tissue distribution [23,31]. These mechanisms are not mutually exclusive and may operate simultaneously depending on the compound identity, formulation, dose, application route, exposure time, plant species, and culture system.

Among these bioactive inputs, chitosan and chitosan-based nanoparticles have gained increasing attention because they combine biodegradability, biocompatibility, film-forming capacity, antimicrobial activity, and elicitor-like effects in plants [32]. In agricultural and plant-based systems, chitosan has been investigated as a seed coating, foliar treatment, postharvest edible coating, plant defense elicitor, and carrier for controlled delivery of nutrients, pesticides, biocontrol agents, or bioactive compounds [33].

This multifunctional nature makes elicitors highly valuable from a biotechnological perspective, but also difficult to interpret from a regulatory perspective. In scientific literature, compounds such as jasmonates, salicylates, chitosan derivatives, microbial metabolites, and nanomaterial-based systems are often described according to their biological role as elicitors, stress modulators, or growth-promoting agents [22,34]. In regulatory practice, however, classification depends less on biological mechanism alone and more on the declared use, product formulation, application context, exposure scenario, and label claim [35,36]. As a result, the same substance may be interpreted differently across jurisdictions, potentially falling within frameworks for fertilizing products, plant biostimulants, plant protection products, biofertilizers, bioinputs, or process aids [37]. Chitosan illustrates this regulatory ambiguity particularly well: it is widely described in the scientific literature as an elicitor and biostimulant-like material, yet specific chitosan forms and claims may also be interpreted under plant protection-related frameworks depending on the intended use [38].

This complexity is further amplified when elicitors are incorporated into encapsulated, controlled-release, or nano-enabled delivery systems. These formulations can improve stability, uptake, and delivery efficiency, but they may also modify persistence, bioavailability, residue profiles, environmental fate, and non-target exposure [39]. Under these conditions, the relevant regulatory object is no longer only the active compound, but the complete formulation and its behavior under specific use conditions [40,41]. Consequently, regulatory assessment increasingly requires a formulation-level perspective capable of considering not only biological function, but also physicochemical identity, exposure context, and intended use.

In this sense, the regulatory challenge is closely linked to the growing diversification of bioactive inputs used in plant-based production systems. Substances initially studied as elicitors in plant cell cultures may later be formulated or commercialized as plant biostimulants, microbial products, nano-enabled agricultural inputs, or broader bioinputs, depending on their composition and claim. This creates conceptual and regulatory boundaries that are not always clearly defined, especially when multifunctional products simultaneously affect stress responses, nutrient-related processes, defense signaling, and metabolite accumulation.

Accordingly, the purpose of this review is to analyze the current regulatory status and classification boundaries of elicitors, plant biostimulants, biofertilizer- and bioinput-related products, and nano-enabled inputs used in plant-based systems for the production of pharmaceutically important compounds. This review examines how these materials are interpreted across selected regulatory frameworks in the European Union, the United States, Canada, Brazil, India, China, and Mexico, with particular attention to the distinction between biological function and legal claim. The analysis focuses on how formulation, intended use, exposure scenario, product composition, and label claim influence whether a material is treated as a fertilizing product, plant biostimulant, biofertilizer, bioinput, plant protection product, process input, or nano-enabled formulation. By integrating scientific evidence with official regulatory instruments, this review aims to clarify the main areas of convergence and fragmentation among current frameworks and to identify regulatory gaps affecting multifunctional elicitors and advanced formulations used in plant biofactories and agricultural applications.

By following this approach, the review moves from the general context of plant biofactories toward the more specific regulatory challenges created by multifunctional and novel bioactive inputs. Elicitation is first discussed as a biotechnological strategy to improve specialized metabolite production, before examining how the same biological functions may acquire different legal meanings when translated into commercial products or agricultural formulations. The discussion then progresses from core regulatory definitions to selected international frameworks, with particular attention to how nano-enabled and encapsulated systems further complicate product classification, safety evaluation, and claim substantiation.

## 2. Elicitation Strategies in Plant-Based Production Systems

Building on the role of plant cell biofactories as controlled platforms for producing pharmaceutically important compounds, this section focuses on elicitation as one of the main strategies used to increase and stabilize specialized metabolite production. The productivity of these systems is determined not only by the biological material itself, but also by the process variables used to modulate plant metabolism, including culture conditions, precursor feeding, metabolic engineering, and elicitation [11]. Among these strategies, elicitation occupies a central position because it can activate defense-associated signaling networks and redirect metabolic flux toward alkaloids, terpenoids, phenolics, glycosides, saponins, and other bioactive compounds [18]. Therefore, elicitation represents both a practical tool for improving plant cell, tissue, and organ cultures and a starting point for later regulatory discussions concerning process inputs, product identity, formulation, and claim-dependent classification.

Elicitation can be broadly defined as the controlled exposure of plant cells, tissues, or organs to physical, chemical, or biological stimuli capable of inducing metabolic responses that resemble those triggered during stress, defense, or symbiotic interactions [42]. In plant cell and organ cultures, elicitors are commonly classified as biotic or abiotic. Biotic elicitors include yeast extract, fungal or bacterial cell-wall fragments, microbial culture filtrates, chitosan, oligosaccharides, and pathogen-derived molecules. Abiotic elicitors include jasmonates, salicylates, ultraviolet radiation, heavy metals, osmotic stress, temperature shifts, electrical stimulation, and, more recently, nanomaterials or nano-enabled delivery systems [18]. Studies on plant cell factories emphasize that elicitation has been used to enhance the production of high-value compounds such as taxanes, ginsenosides, aryltetralin lignans, phenolics, and other specialized metabolites, with newer approaches incorporating coronatine, cyclodextrins, and nano-elicitors [43].

The mechanistic principle of elicitation is closely related to the activation of stress-responsive signaling pathways. Jasmonic acid and methyl jasmonate are among the most widely used elicitors because they participate in wound and defense signaling and frequently upregulate genes involved in terpenoid, alkaloid, and phenylpropanoid metabolism [22]. Salicylic acid is also widely used, particularly in systems where defense-like responses, redox regulation, and phenolic metabolism are relevant [44]. Chitosan and chitosan-derived oligosaccharides are especially attractive because they can be perceived by plant cells as pathogen-associated or damage-associated molecular signals, triggering oxidative bursts, defense-related enzymes, phenylpropanoid metabolism, and the accumulation of specialized metabolites [23]. However, elicitor responses are highly system-specific and depend on concentration, exposure time, culture age, cell line, degree of differentiation, nutrient composition, light regime, and the balance between biomass growth and secondary metabolism [45].

A central feature of elicitation is that productivity is not determined only by the presence or absence of a stimulus, but by the optimization of a dose–time–culture window. Excessive elicitor concentrations may inhibit cell proliferation, reduce viability, or redirect cellular resources toward generalized stress responses rather than target metabolite accumulation. Conversely, suboptimal doses may induce only weak transcriptional or metabolic responses. This explains why many successful studies report narrow optimal ranges for elicitor concentration, exposure time, and culture age [26].

From a bioprocess perspective, elicitation must be evaluated critically. Although many studies report large fold increases, fold-change alone can be misleading when basal production is extremely low [18]. Industrial relevance depends on absolute yield, volumetric productivity, extracellular accumulation, extraction efficiency, elicitor cost, culture stability, batch-to-batch reproducibility, and compatibility with downstream purification [12]. Several studies emphasize that plant cell and organ cultures offer year-round production under controlled conditions but also note that claims of commercial feasibility should be assessed carefully because scale-up, genetic stability, productivity, and process economics remain persistent challenges [11]. In this regard, paclitaxel production by *Taxus* cell cultures remains one of the most frequently cited examples of successful industrial implementation, while many other metabolite systems remain at laboratory or pilot scale [46].

This limitation can be illustrated by well-known elicitation systems. In *Taxus wallichiana* cell suspension cultures, methyl jasmonate has been evaluated not only in flasks but also during bioreactor cultivation, including 20 L bubble-type bioreactors and subsequent upscaling to 75 L bioreactors. Although MeJA enhanced taxoid accumulation, the response depended on culture age, subculture history, cultivation cycle, and the taxoid profile, with older cultures showing a predominance of C14-hydroxylated taxoids rather than consistent accumulation of paclitaxel (https://doi.org/10.3390/biom13060969). Additionally, the use of dimethyl-β-cyclodextrin (DIMEB), alone or combined with MeJA, has been demonstrated to enhance extracellular trans-resveratrol accumulation in *Vitis vinifera* cell suspensions. This system was optimized in 250 mL shaken flasks and then tested in a 2 L stirred bioreactor, where production depended on biomass density, DIMEB-to-biomass ratio, order of elicitor addition, aeration, agitation, temperature, and sucrose concentration (https://doi.org/10.3390/biom13101529). This indicates that an elicitor effective at a small scale may not ensure stable production of the desired compound during prolonged maintenance or larger-scale operation. 

Therefore, elicitation should be understood as a controllable but highly context-dependent intervention. Its relevance for pharmaceutically important compounds lies in its ability to activate latent or weakly expressed biosynthetic pathways, increase precursor flux, induce transcription factors, promote metabolite secretion, and modify the bioactive profile of plant-derived cultures [18,47]. At the same time, elicitation introduces questions that extend beyond productivity. The identity, safety profile, persistence, and destination of the elicitor may influence how the resulting process or product is assessed, particularly when elicitors remain as residues, alter extract composition, or are incorporated into advanced formulations. These considerations provide the transition to the following section, where the distinction between elicitors and plant biostimulants is examined in terms of scientific function, product claims, and regulatory classification.

## 3. From Elicitors to Biostimulants: Scientific Functions Versus Legal Claims

The previous section showed that elicitation is a powerful strategy for activating specialized metabolism in plant cells, tissues, and organs. However, the same biological properties that make elicitors valuable in controlled biofactory systems also complicate their interpretation when they are incorporated into agricultural products, commercial formulations, or scalable production processes. A compound used in vitro to stimulate alkaloid, terpenoid, phenolic, or glycoside biosynthesis may also improve nutrient uptake, enhance abiotic stress tolerance, induce defense responses, alter hormonal balance, increase crop quality, or contribute to pathogen suppression, depending on the biological system and application context [48]. This multi-functionality creates a regulatory challenge because scientific categories are usually based on mechanisms of action, whereas legal categories are generally determined by intended use, label claim, product composition, formulation, and exposure scenario.

Plant biostimulants emerged as a category intended to distinguish products that improve plant performance through mechanisms other than direct nutrient supply or direct pest control. In the literature, plant biostimulants have been described as substances or microorganisms applied to plants with the aim of enhancing nutrition efficiency, abiotic stress tolerance, and/or crop quality traits, independently of their nutrient content [34]. This definition helped separate biostimulants from fertilizers, which primarily supply nutrients, and from plant protection products, which are associated with pest or disease control. Earlier works also showed that the term “biostimulant” had historically been used inconsistently, ranging from non-fertilizer growth-promoting products to hormone-containing substances or natural preparations with broad biological activity [49]. This historical ambiguity is critical because many elicitors produce physiological responses that overlap with what is now described as biostimulant activity.

From a physiological perspective, the transition from elicitor to biostimulant is understandable. Many elicitors activate stress perception, reactive oxygen species signaling, phytohormone networks, antioxidant enzymes, phenylpropanoid metabolism, and secondary metabolite biosynthesis [50]. These responses may support improved stress adaptation, enhanced quality traits, or increased accumulation of bioactive phytochemicals, particularly when the treatment is applied under appropriate dose and timing conditions [31]. Therefore, the same biological mechanism that is useful for increasing a pharmaceutically important compound in a plant cell culture may also be interpreted, in an agricultural context, as a biostimulant-like effect.

The European Union provides one of the clearest examples of how this distinction has been formalized in law. Regulation (EU) 2019/1009 defines a plant biostimulant as an EU fertilizing product whose function is to stimulate plant nutrition processes independently of the product’s nutrient content, with the sole aim of improving nutrient use efficiency, tolerance to abiotic stress, quality traits, or the availability of confined nutrients in the soil or rhizosphere [51]. This definition does not classify biostimulants primarily according to chemical origin or biochemical mechanism, but according to the type of beneficial effect claimed for the plant or rhizosphere. Thus, a material with elicitor-like activity may be considered within a biostimulant-type framework when the declared claim concerns nutrition-related processes, abiotic stress tolerance, or quality traits. Nevertheless, the same material may fall outside that framework if the claim shifts toward pathogen control, disease suppression, or antimicrobial activity.

This claim-based approach has practical advantages because it allows regulators to evaluate products according to their declared function. However, it can be insufficient to capture the full complexity of multifunctional elicitors. Chitosan illustrates this difficulty particularly well. Scientifically, chitosan and its derivatives have been described as materials with protective, biostimulating, and eliciting effects in plants, capable of influencing growth, defense responses, oxidative signaling, antimicrobial activity, and yield quality [31]. Recent evidence also indicates that chitosan responses are dose, tissue, and system-dependent; low to moderate concentrations may support growth-related responses, whereas higher concentrations can inhibit root development or reinforce growth–defense trade-offs [52]. This multifunctionality makes it difficult to assign chitosan-like compounds to a single regulatory category without considering formulation, dose, application site, target effect, and claim.

This claim-dependent classification becomes even more relevant for novel chitosan nanoformulations that can change the role of the material from a free elicitor to a seed or fruit coating, nanocarrier, controlled-release system, antimicrobial formulation, or plant protection-related product [53]. Therefore, chitosan-based nanosystems reinforce the need to distinguish between the biological function of the material and the legal claim attached to the final formulation.

Chitosan is also useful as a regulatory example because its classification can shift according to use. In the European Union, chitosan hydrochloride has been approved as a basic substance under Regulation (EC) No 1107/2009 for plant protection-related uses through Commission Implementing Regulation (EU) No 563/2014, and Commission Implementing Regulation (EU) 2021/1446 later corrected its CAS number, highlighting the importance of precise substance identity for regulatory certainty [38]. However, this basic-substance approval does not automatically constitute authorization as a plant biostimulant. Under Regulation (EU) 2019/1009, a chitosan-containing product may be placed on the market as a non-microbial plant biostimulant only if it complies with the requirements for EU fertilizing products, particularly Annex I, Product Function Category 6(B), as well as the applicable component material, conformity assessment, labeling, and claim-substantiation provisions [51]. Thus, chitosan does not support automatic simultaneous dual-category registration; rather, each regulatory route must be justified independently according to formulation, intended use, exposure scenario, and label claim. This example illustrates a central point for this review: a molecule may be scientifically described as an elicitor and may show biostimulant-like effects, but if the intended claim concerns protection against harmful organisms or disease suppression, the product may move toward a plant protection framework rather than a fertilizing product framework R1-2.

The boundary with plant protection products is therefore particularly important. Under Regulation (EC) No 1107/2009, plant protection products include products containing active substances, shields, or synergists and intended to protect plants or plant products against harmful organisms, influence plant life processes other than nutrients, preserve plant products, destroy undesired plants, or prevent undesired growth [54]. Consequently, an elicitor-based product claiming improved nutrient use efficiency or abiotic stress tolerance may be positioned as a plant biostimulant, whereas a similar product claiming anti-fungal activity, pathogen suppression, or induced resistance against disease may be interpreted as a plant protection product [24]. This does not mean that the biological mechanism has changed; rather, the regulatory meaning of the product changes because the declared use has changed.

A similar claim-dependent logic appears in the United States. The U.S. Environmental Protection Agency (EPA) has issued guidance to clarify which plant biostimulants, biological substances, mixtures, and associated label claims may be considered plant regulators and therefore subject to regulation as pesticides under the Federal Insecticide, Fungicide, and Rodenticide Act (FIFRA) [36]. This reinforces the idea that the regulatory boundary is not defined exclusively by whether a substance is natural, biological, microbial, or chemically simple. Instead, classification depends on whether the product’s label, marketing, composition, and intended use imply plant regulation, pest control, disease suppression, or another regulated pesticidal function.

For elicitors used in plant cell biofactories, this creates an additional layer of ambiguity. In a contained bioreactor, methyl jasmonate, salicylic acid, chitosan, cyclodextrins, or microbial-derived signals may be better interpreted as process inputs used to activate specialized metabolism and improve the yield of purified pharmaceutical compounds [26]. In contrast, when similar compounds are applied to crops or marketed as agricultural products, they may be evaluated as biostimulants, plant protection products, basic substances, biofertilizers, biofungicides, or plant growth regulators, depending on the jurisdiction and claim [55]. Thus, the regulatory status of an elicitor is not intrinsic to the molecule alone; it emerges from the interaction between material identity, formulation, biological effect, application context, exposure scenario, and declared purpose [56].

This creates a conceptual gap for compounds with multiple biological effects. Legal definitions are necessary for market authorization, labeling, efficacy testing, and consumer protection, but they tend to reduce multifunctional biological responses into a limited number of claim categories. In the EU framework, plant biostimulant claims are structured around nutrient use efficiency, abiotic stress tolerance, quality traits, and nutrient availability, while plant protection claims are associated with harmful organisms and disease control [51]. However, elicitors frequently operate across this boundary because defense signaling, abiotic stress responses, redox regulation, hormone crosstalk, and specialized metabolism are biologically interconnected. A treatment that increases phenolics or alkaloids may simultaneously improve antioxidant capacity, modify pathogen resistance, alter quality traits, and affect stress tolerance [48].

This limitation becomes more evident when elicitors are incorporated into advanced formulations. Encapsulated elicitors, chitosan nanoparticles, cyclodextrin complexes, polymeric carriers, and nano-enabled systems may change solubility, uptake, persistence, release kinetics, and tissue distribution [40,41]. As a result, the regulatory object may shift from the elicitor molecule alone to the complete formulation, its mode of delivery, and its exposure profile. A free elicitor used transiently in a plant cell suspension culture may pose a different regulatory question from the same elicitor formulated as a foliar nanocarrier, soil-applied input, or controlled-release agricultural product [47]. Therefore, classification cannot rely exclusively on biochemical function or on a single claimed effect. Instead, it requires a broader understanding of how regulatory frameworks define fertilizers, biofertilizers, biostimulants, and bioinputs, and how these definitions determine the boundary between nutrition, stress modulation, plant protection, and biotechnological process inputs [34].

## 4. Regulatory Definitions of Fertilizers, Biofertilizers, Plant Biostimulants and Bioinputs

Because the regulatory interpretation of elicitor-like products depends strongly on declared function, formulation, use context, and label claim, a clear understanding of the main legal categories is required before comparing specific jurisdictions. Therefore, this section focuses on how key regulatory categories are defined and delimited. Rather than revisiting the biological mechanisms of these compounds, the aim is to clarify how fertilizers, biofertilizers, plant biostimulants, and bioinputs are positioned within selected regulatory frameworks and why these definitions are relevant for multifunctional bioactive inputs used in plant-based production systems.

Fertilizer regulation has traditionally been associated with the direct provision of nutrients to plants [57]. This nutrient-centered approach remains visible in frameworks such as Canada’s Fertilizers Act, where a fertilizer is defined as a substance or mixture containing nitrogen, phosphorus, potassium, or other plant food manufactured, sold, or represented for use as a plant nutrient [58]. In the same framework, supplements are distinguished from fertilizers because they are represented for improving soil physical condition or aiding plant growth or crop yield, without necessarily acting as direct nutrient sources [59]. This distinction is important because it creates a regulatory space for products that influence plant performance but do not fit within the conventional fertilizer definition.

The European Union has adopted a broader structure through Regulation (EU) 2019/1009, which organizes EU fertilizing products into Product Function Categories. This framework includes fertilizers, liming materials, soil improvers, growing media, inhibitors, plant biostimulants, and fertilizing product blends [51]. Under this regulation, plant biostimulants are classified within the fertilizing product framework, but their legal function is not defined by nutrient content. Instead, a plant biostimulant is an EU fertilizing product whose function is to stimulate plant nutrition processes independently of the product’s nutrient content, with the sole aim of improving nutrient use efficiency, tolerance to abiotic stress, quality traits, or the availability of confined nutrients in the soil or rhizosphere [49]. This definition is central for regulatory classification because it links the product category to specific permitted claims rather than to a single chemical or biological mechanism.

The EU framework also distinguishes between microbial and non-microbial plant biostimulants. Microbial plant biostimulants are subject to specific component material requirements, and only certain microorganism groups are currently covered under the harmonized positive-list approach, including *Azotobacter* spp., mycorrhizal fungi, *Rhizobium* spp., and *Azospirillum* spp. [60]. This is relevant because microbial products with experimentally demonstrated plant-growth-promoting, elicitor, or stress-modulating activity may remain outside the harmonized EU route if their microorganisms are not included in the corresponding component material categories [61].

For microbial products whose active microorganisms are not included in this EU framework, the harmonized European marked route under Regulation 2019/1009 is currently not available as a microbial plant biostimulant. This limitation may change only if the corresponding microorganism, strain, or additional processing method is incorporated into Annex II through a delegated act, after verification of identity, safety, agronomic efficiency, production process, residual metabolites, and environmental behavior [51]. Therefore, these products are generally placed on the market through national fertilizer, biostimulant, or related input regulations of individual Member States. However, this does not necessarily restrict them to a single national market, since a product lawfully marketed in one Member State may seek access to other Member States under the EU mutual recognition framework established by Regulation (EU) 2019/515 [62]. In addition, if the microbial product makes claims related to protection against harmful organisms, disease suppression, or biocontrol, it may move outside the fertilizing product framework and require assessment under plant protection legislation, particularly Regulation (EC) No 1107/2009 [54]. Thus, non-classified microbial elicitors remain regulated through non-harmonized or claim-dependent pathways rather than through the harmonized EU plant biostimulant route. R1-3.

Therefore, these products are generally placed on the market through national fertilizer, biostimulant, or related input regulations of individual Member States. However, this does not necessarily mean that they are restricted to a single national market. Once a product is lawfully marketed in one Member State, access to other Member States may be sought through the EU mutual recognition framework under Regulation (EU) 2019/515, although competent authorities may restrict marketing where justified by public health, safety, or environmental protection considerations. In addition, if the microbial product makes claims related to protection against harmful organisms, disease suppression, or biocontrol, it may move outside the fertilising product framework and require assessment under plant protection legislation. Thus, non-CMC 7 microbial elicitors remain regulated through non-harmonized or claim-dependent pathways rather than through the harmonized EU plant biostimulant route.

The transition from legal definition to claim substantiation is another important regulatory development. The EN 17700 series, published in 2024, provides standardized guidance for demonstrating plant biostimulant claims. EN 17700-1:2024 establishes general principles for claim justification, whereas the other parts address specific claim categories such as nutrient use efficiency, abiotic stress tolerance, quality traits, and nutrient availability [63]. EN 17700-3:2024 specifically addresses claims related to tolerance to abiotic stress and should be applied together with EN 17700-1:2024 and EN 17724:2024 on plant biostimulant terminology [64]. These standards are relevant because a claim-based regulatory category requires experimental evidence showing that the declared function is achieved under appropriate conditions.

Biofertilizers follow a more specific regulatory logic because they are generally associated with living microorganisms or microbial consortia that improve nutrient availability, acquisition, or transformation rather than directly supplying nutrients in the same way as conventional fertilizers [65,66]. Their agronomic functions commonly include biological nitrogen fixation, phosphorus solubilization, potassium or zinc mobilization, siderophore production, mycorrhizal nutrient uptake, and stimulation of nutrient cycling in the rhizosphere [67]. Therefore, biofertilizers occupy an intermediate position between nutrient-centered fertilizer regulation and broader biological input frameworks. Regulatory definitions, however, may be narrower than scientific usage. For example, India’s Fertilizer Control Order has traditionally defined biofertilizers as carrier-based solid or liquid products containing living microorganisms that are agriculturally useful for nitrogen fixation, phosphorus solubilization, or nutrient mobilization [68]. In contrast, India’s more recent biostimulant framework, introduced through Notification S.O. 882(E), covers substances, microorganisms, or combinations thereof that stimulate physiological processes and enhance nutrient uptake, growth, yield, nutrient efficiency, crop quality, or stress tolerance, while excluding pesticides and plant growth regulators regulated under the Insecticides Act [69]. This distinction is relevant for microbial elicitors because a microorganism may be scientifically described as plant-growth-promoting, stress-modulating, or elicitor-active, but its regulatory classification depends on whether the declared function is nutrient mobilization, biostimulation, plant protection, or another claim-dependent use.

In the United States, the definitional landscape remains more fragmented. Plant biostimulants intersect with fertilizer, soil amendment, beneficial substance, microbial inoculant, plant regulator, and pesticide-related frameworks. The 2019 USDA Report to Congress discussed possible definitions and regulatory options for plant biostimulants, whereas EPA’s draft guidance focused on clarifying which products and claims may fall within plant regulator or pesticide regulation under FIFRA [70]. This differs from the EU approach because the United States has not established a single harmonized federal plant biostimulant category. Instead, the regulatory route depends strongly on label claims, market representation, and whether the product implies plant regulation or pesticidal activity.

The term bioinput represents a broader regulatory concept. Brazil’s Law No. 15.070/2024 defines bioinput as a product, process, or technology of plant, animal, or microbial origin, including those derived from biotechnological processes or structurally similar and functionally identical to natural-origin products, intended for use in the production, protection, storage, or processing of agricultural products, or in aquatic and planted forest production systems [71,72]. This definition is broader than the plant biostimulant concept because it includes not only products but also processes and technologies. Such breadth may be useful for multifunctional biological and biotechnological inputs, although it also requires technical sub-classification to avoid grouping products with different functions, exposure profiles, and risk levels under a single general category.

Other jurisdictions maintain more category-specific frameworks. In Mexico, for example, the regulatory language has traditionally focused on sanitary registration of pesticides and plant nutrients rather than on a harmonized plant biostimulant or bioinput category. NOM-182-SSA1-2010 establishes labeling requirements for plant nutrients, whereas pesticide-related products follow separate regulatory routes [73]. This creates potential ambiguity for elicitor-like substances, microbial extracts, chitosan derivatives, jasmonates, salicylates, or nanoformulated inputs when their declared function is not direct nutrient supply but metabolic activation, stress modulation, quality enhancement, or plant response regulation.

Overall, these definitions show that regulatory categories are operational tools rather than simple scientific descriptors. They determine the evidence required for authorization, the claims that can be made, the safety assessments that may be triggered, the labeling language permitted, and the route through which products enter the market. Fertilizers are generally linked to nutrient supply; biofertilizers to microbial nutrient functions; plant biostimulants to claim-based improvements in nutrient use, abiotic stress tolerance, quality traits, or nutrient availability; and bioinputs to a broader biological or biotechnological scope. For multifunctional elicitors and advanced formulations, the central challenge is that one product may interact with several of these categories simultaneously, making classification dependent on claim, formulation, composition, and exposure context.

## 5. Global Regulatory Landscape: EU, USA, Canada, Brazil, India, China and Mexico

The previous section established that fertilizers, biofertilizers, plant biostimulants, and bioinputs are not interchangeable categories but regulatory constructs shaped by function, composition, evidence requirements, and intended use. Building on that definitional basis, this section compares how selected jurisdictions translate these categories into regulatory practice. This comparison is particularly relevant for multifunctional elicitors because the same compound, microorganism, extract, or formulation may be interpreted differently when it moves from a contained plant cell biofactory to a commercial agricultural product or a field-applied input. Rather than reviewing each country’s entire fertilizer or pesticide legislation, this section focuses on the regulatory instruments most relevant to elicitors, biostimulants, biofertilizers, bioinputs, and related bioactive formulations.

The European Union currently provides one of the most structured supranational frameworks for plant biostimulants. Regulation (EU) 2019/1009 established harmonized rules for making EU fertilizing products available on the market and introduced plant biostimulants as a specific Product Function Category within the broader fertilizing products framework. Under this system, a CE-marked fertilizing product may circulate within the EU market when it complies with the applicable requirements, although the regulation does not fully replace national rules for products marketed outside the harmonized EU route [51]. For plant biostimulants, the regulatory focus is not primarily the chemical origin or biological nature of the product, but whether the claimed function falls within the recognized scope of stimulating plant nutrition processes to improve nutrient use efficiency, abiotic stress tolerance, quality traits, or nutrient availability in the soil or rhizosphere [74]. This makes the EU framework especially relevant for elicitor-like products: stress- or quality-related claims may be compatible with a biostimulant route, whereas claims centered on pathogen suppression, pest control, or disease resistance may move the product toward plant protection legislation.

A major strength of the EU approach is the movement from broad legal definition toward formal claim substantiation. The EN 17700 series, published in 2024, provides a structured basis for demonstrating plant biostimulant claims, including general principles and specific guidance for nutrient use efficiency, abiotic stress tolerance, quality traits, and nutrient availability [63]. EN 17700-2:2024 addresses nutrient use efficiency claims [75], while EN 17700-3:2024 addresses abiotic stress tolerance claims and includes trial design, agronomic markers and stress markers such as antioxidant responses, reactive oxygen species, gene expression, metabolites, relative water content, chlorophyll fluorescence and lipid peroxidation [64]. This represents an important regulatory maturation: biostimulant classification is no longer only a matter of wording, but also of experimental evidence supporting the declared claim.

In the United States, the regulatory landscape remains more fragmented because there is no single federal plant biostimulant regulation equivalent to Regulation (EU) 2019/1009. Instead, classification depends strongly on whether the product or its claims fall under fertilizer, soil amendment, beneficial substance, plant regulator, or pesticide-related frameworks [76]. The U.S. Environmental Protection Agency has issued guidance to clarify which plant biostimulants, biological substances, mixtures, and associated label claims may be considered plant regulators and therefore subject to regulation as pesticides under the Federal Insecticide, Fungicide, and Rodenticide Act [36]. This approach is particularly relevant for elicitors such as jasmonates, salicylates, chitosan derivatives, microbial signals, or other stress-modulating compounds, because claims implying plant regulation, pest suppression, or disease control may trigger a different regulatory route from claims related to general plant performance.

At the same time, the United States has moved toward greater terminology alignment at the state level. In 2024, the Association of American Plant Food Control Officials approved model language for beneficial substances and plant biostimulants, and California subsequently introduced definitions for “beneficial substance” and “plant biostimulant” through Senate Bill 1522 [77,78]. The California Department of Food and Agriculture later clarified the conditions under which plant biostimulant claims may appear on fertilizing material labels. These developments suggest a gradual movement toward more consistent terminology, but the U.S. system remains claim-sensitive and partly state-dependent.

Canada follows a different model based on the Fertilizers Act and Fertilizers Regulations. Some fertilizers and most supplements require mandatory pre-market assessment and registration before importation or sale in Canada [59]. This regulatory status of a product, depending on how it is represented in the marketplace, makes Canada conceptually like the EU and the USA in one key aspect: product claims and market representation are decisive for classification.

Canada is also notable because microbial supplements are explicitly addressed. CFIA’s 2024 trade memorandum T-4-109 states that microbial supplements represented to contain microorganisms as active ingredients require registration prior to importation and sale in Canada; this includes pure cultures of bacteria or fungi, microbial consortia and genetically modified microorganisms, including those generated through gene editing or synthetic biology [79]. This shows that microbial biostimulants and microbial elicitors are evaluated not only through their agronomic function, but also through identity, composition, safety, labeling and registration status.

Brazil has recently taken one of the most expansive approaches by adopting a dedicated bioinputs framework. Law No. 15,070 in 2024 regulates the production, importation, exportation, registration, commercialization, use, inspection, research, experimentation, packaging, labeling, advertising, transport, storage, service provision, waste and packaging disposal, and incentives to produce bioinputs for agricultural, livestock, aquaculture and forestry use [72]. This is broader than the EU biostimulant framework because it does not restrict the regulatory concept to plant nutrition processes. Instead, it recognizes bioinputs as a broad category of biological or biotechnological products, processes and technologies relevant to production and protection systems.

Brazil’s approach is particularly important for multifunctional elicitors because a broad bioinput category may better accommodate microbial inoculants, biofertilizers, biostimulants, biocontrol products and other biologically derived inputs. However, broadness also creates a need for detailed secondary regulation. In 2026, Brazil’s Ministry of Agriculture and Livestock opened a proposal for a decree regulating Law No. 15,070/2024, covering the full life cycle of bioinputs, including research, experimentation, registration, production, importation, exportation and use [80]. Therefore, Brazil represents a regulatory model that is conceptually flexible, but still dependent on technical implementation rules to avoid grouping products with very different functions, risks and evidence requirements under the same general label.

India has moved from a provisional and highly populated biostimulant market toward a more controlled schedule-based system. Biostimulants were incorporated into the Fertilizer Control Order through the 2021 regulatory framework, but the government later decided not to extend provisional registrations beyond June 2025 [68]. According to the Press Information Bureau, provisional registrations became invalid, and only 146 biostimulant products included in Schedule VI of the Fertilizer Control Order remained valid [69]. This shift is significant because provisional registration had allowed more than 8000 products to remain temporarily on the market while manufacturers generated bioefficacy, toxicity and chemistry data required for inclusion in Schedule VI. Compared with the EU, which has harmonized plant biostimulants within fertilizing products, India has integrated biostimulants into an existing fertilizer control system and now requires products to be specifically included in Schedule VI. This reduces the space for loosely defined commercial products and increases the importance of demonstrating bioefficacy, composition and safety.

China does not currently appear to have a separate plant biostimulant regulation equivalent to the EU model. Biostimulant-like products are generally handled under fertilizer or pesticide-related frameworks depending on composition and claims. China’s fertilizer registration system is administered by the Ministry of Agriculture and Rural Affairs, and the 2022 version of the Fertilizer Registration Management Measures identifies MARA as responsible for national fertilizer registration, filing and supervision [81]. Applicants must provide information related to product chemistry, fertilizer efficiency, safety and labeling, and standardized field trials in China are required before registration.

Recent updates suggest that China is strengthening efficacy documentation. In October 2025, MARA’s government service platform reportedly updated the fertilizer registration guidance with clearer requirements for fertilizer efficiency experiments, including stronger expectations for trial leadership, video records and field experiment documentation [82]. China also recognizes microbial fertilizers through agricultural standards, including NY/T 1113-2006 on microbial fertilizer terminology [83]. Thus, China’s current approach is not centered on a harmonized “plant biostimulant” category but on fertilizer registration, microbial fertilizer standards and claim-dependent movement into other regulatory frameworks.

Mexico currently regulates products relevant to this review primarily through the categories of pesticides and plant nutrients rather than through a formal plant biostimulant or bioinput category. Comisión Federal para la Protección contra Riesgos Sanitarios (COFEPRIS) maintains the sanitary registration system for pesticides and plant nutrients, and its public consultation platform allows users to search for sanitary registrations for pesticides, plant nutrients and maximum residue limits. NOM-182-SSA1-2010, which regulates the labeling of plant nutrients, remains listed as active by COFEPRIS [73]. NOM-077-FITO-2000 establishes the requirements and specifications for biological effectiveness studies of plant nutrition inputs, and the 2011 modification identifies plant nutrition inputs as substances or mixtures containing useful elements for plant nutrition and development [84].

For elicitor-like products, the Mexican framework therefore remains strongly dependent on whether the product is positioned as a plant nutrient, fertilizer-like input or pesticide. This creates ambiguity for substances such as chitosan, salicylic acid, jasmonates, microbial extracts or nanoformulated elicitors, which may not fit comfortably into the plant nutrient category if their primary effect is metabolic activation, stress signaling or defense induction.

These regulatory systems show both convergence and fragmentation. The main point of convergence is that most jurisdictions increasingly require some combination of product identity, label clarity, efficacy substantiation, safety information, and, in some cases, pre-market registration. The strongest divergence lies in the regulatory entry point. The EU treats plant biostimulants as a harmonized fertilizing product category supported by claim-specific substantiation. The USA remains divided between federal pesticide oversight and state-level fertilizer or beneficial-substance frameworks. Canada regulates many biostimulant-like products as fertilizers or supplements, with a strong emphasis on pre-market assessment and microbial supplement registration. In contrast, other jurisdictions like Brazil, India, and Mexico have followed more heterogeneous routes, ranging from broad bioinput frameworks and schedule-based authorization systems to fertilizer/pesticide-based approaches that still lack a dedicated biostimulant or bioinput category.

These differences are not merely administrative. They determine which claims can be made, what evidence must be generated, whether microbial or gene-edited components trigger additional requirements, how products are labelled, and whether multifunctional elicitors are treated as nutrition-related inputs, microbial products, plant protection substances, bioinputs, or process aids. For plant cell biofactories, this global variability is especially relevant because elicitors may be used under contained conditions as process inputs, while similar materials may also be marketed as agricultural formulations in open environments.

Table 1 summarizes the main regulatory entry points, product categories, evidence requirements, and classification implications for elicitor-like, biostimulant-like, microbial, and nano-enabled inputs in the selected regions. The comparison shows that most jurisdictions converge around product identity, labelling, efficacy, and safety, but differ substantially in the legal category through which products enter the market and in the extent to which multifunctional or advanced formulations are explicitly addressed.

This comparison shows that the main differences lie in the legal entry route, the role of product claims, and the existence or absence of dedicated categories for biostimulants, bioinputs, microbial products, and nano-enabled formulations.

## 6. Nano-Enabled and Encapsulated Elicitors: Emerging Regulatory Concerns

The comparison of international frameworks shows that most regulatory systems still classify agricultural and plant-related inputs mainly according to product function, composition, intended use, and label claim. However, this claim-based logic becomes more difficult to apply when elicitors are incorporated into nano-enabled, encapsulated, or controlled-release delivery systems. In these cases, the regulatory object is no longer only the active molecule but the complete formulation, including the carrier, particle size distribution, surface chemistry, encapsulation efficiency, release profile, degradation behavior, persistence, and exposure pathway [40,85]. This distinction is particularly relevant for plant cell biofactories and agricultural systems because nano-enabled elicitors may be used either as contained process inputs under controlled bioreactor conditions or as field-applied products with biostimulant-, biofertilizer-, or plant-protection-like claims [86].

Nano-enabled elicitors and encapsulated formulations have attracted increasing attention because they can improve the solubility, stability, uptake, targeted delivery, and controlled release of different bioactive compounds [87]. In agricultural systems, nanocarriers have been proposed to increase the efficiency of agrochemicals, reduce losses, improve delivery to target tissues, and decrease the total dose required to achieve a biological response [88,89]. These properties are also relevant for elicitation because many elicitors require precise control of dose, timing, and exposure duration to activate specialized metabolism without compromising growth or cell viability [19]. In this sense, encapsulation and nanoscale delivery may provide a technological route to convert transient elicitor exposure into more controlled and reproducible metabolic stimulation.

**Figure 2:**
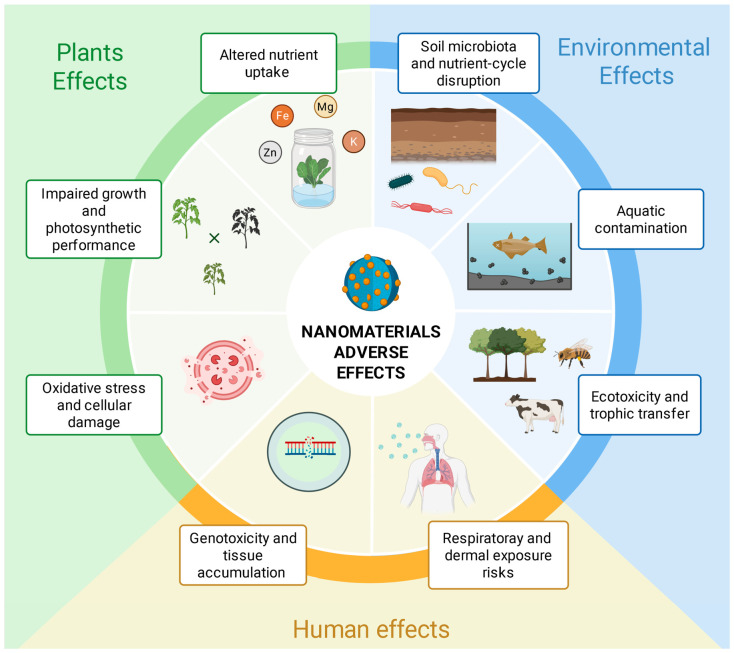
Potential adverse effects of nanomaterials on plants, the environment, and human health. Created in BioRender (https://www.biorender.com/).

Additionally, the potential risks associated with nano-enabled agricultural inputs are not restricted to their direct effects on treated plants. Once released into agricultural or production systems, nanomaterials may interact with plant tissues, soil particles, microbial communities, water bodies, animals, and humans through different exposure routes [90]. As summarized in Figure 2, these interactions may result in adverse effects at three interconnected levels: plant performance, environmental stability, and human health. Therefore, the regulatory assessment of nano-enabled elicitors should consider not only their intended biotechnological or agronomic function, but also their possible environmental fate, ecotoxicological behavior, and human exposure pathways.

Chitosan nanoparticles provide one of the clearest examples of this dual technological and regulatory relevance. Chitosan is already widely described as a plant defense elicitor, stress modulator, biodegradable polymer, film-forming material, antimicrobial agent, and possible biostimulant-like input [31]. When formulated as nanoparticles, its functional profile may further expand because chitosan nanoparticles can be used as seed coatings, foliar treatments, postharvest edible coatings, carriers for fertilizers or pesticides, delivery systems for biocontrol agents, and elicitors for specialized metabolite production [91]. These applications are particularly attractive because chitosan nanoparticles have been demonstrated to improve dispersion, surface adhesion, bioavailability, controlled release, and interaction with plant tissues compared with bulk chitosan [92].

Several studies illustrate the increasing relevance of chitosan nanoparticles in plant systems. In wheat, chitosan nanoparticles promoted seed germination and seedling growth at lower concentration than bulk chitosan, an effect associated with stronger adsorption to the seed surface, modulation of auxin-related gene expression, and changes in indole-3-acetic acid biosynthesis and transport [93]. In medicinal plant cell cultures, chitosan nanoparticles have also been evaluated as elicitors of secondary metabolism. For example, in *Dracocephalum polychaetum* cell suspension cultures, low and moderate doses of chitosan nanoparticles enhanced the accumulation of phenolic and flavonoid compounds, whereas higher concentrations increased oxidative stress markers such as MDA and H_2_O_2_ [94]. Chitosan-based nanoformulations have also been reported in stress-mitigation studies; chitosan–fulvic acid nanoparticles and chitosan-loaded zinc oxide nanoparticles have been investigated as tools to improve drought tolerance in maize through antioxidant, physiological, and transcriptional responses [33,95].

From a regulatory perspective, this diversity of applications makes chitosan nanoparticles difficult to classify through conventional categories. Therefore, evaluation should not focus only on the presence of chitosan as a known biodegradable polymer, but also on formulation-specific properties such as molecular weight, degree of deacetylation, particle size distribution, surface charge, encapsulation efficiency, release profile, persistence, plant uptake, and non-target exposure [93,94].

The same conceptual issue applies to cyclodextrin complexes, polymeric nanocapsules, lipid-based carriers, metal oxide nanoparticles, nanoemulsions, and nanocomposites. These systems can enhance elicitor delivery, protect active molecules from degradation, improve compound stability, promote metabolite secretion, or enable slow release. However, they may also modify bioavailability, tissue distribution, uptake routes, persistence, transformation, residue potential, and interactions with plant or soil matrices [41]. Consequently, the regulatory concern is not limited to determining whether the original elicitor is known or considered safe. It also requires assessing whether the nanoformulated version generates a distinct exposure scenario, modifies biological interactions, or changes the fate of residues in the final extract, crop, soil, water, or production system [96].

In this context, the European Commission’s 2022 recommendation provides a useful cross-reference for identifying materials that may require specific consideration. It defines nanomaterials as materials composed of solid particles in which 50% or more of the particles in the number-based size distribution fulfil nanoscale criteria [97]. Although this recommendation is not specific to elicitors or plant cell biofactories, it provides a technical basis for recognizing when particle-based formulations may require nanospecific characterization.

Recent regulatory and scientific analyses show that this concern is no longer theoretical. A 2024 report prepared under the European Union Observatory for Nanomaterials reviewed nanomaterial-based and nano-enabled plant protection, biocidal, and fertilizing products for agriculture. The report retrieved 3052 relevant scientific and grey-literature documents and identified knowledge gaps related to applications, exposure, and hazards of nanomaterials and nano-agrochemicals [98]. It also concluded that nano-agrochemicals may offer enhanced performance, but that the EU Plant Protection Products Regulation and Fertilizing Products Regulation still lack specific provisions for nanoforms, unlike the EU Biocidal Products Regulation; this distinction is highly relevant for elicitor-based products because many nano-enabled formulations may not be intended primarily as biocides, yet they may still enter plant tissues, soil systems, water compartments, or the food and pharmaceutical production chain.

The Biocidal Products Regulation provides a useful example of how nanospecific provisions can be incorporated into a sectoral regulatory framework. Regulation (EU) No 528/2012 defines a nanomaterial as an active or non-active substance containing particles, in an unbound state or as aggregates or agglomerates, where 50% or more of the particles in the number-size distribution have one or more external dimensions in the 1–100 nm range [99]. It also establishes that the approval of an active substance does not cover nanomaterials unless this is explicitly mentioned, that risks to human health, animal health, and the environment must be assessed separately when nanomaterials are used in a biocidal product, that products containing nanomaterials are excluded from the simplified authorization procedure, and that labels must identify nanomaterials and include the word “nano” in brackets, together with any specific related risks [99].

However, several investigations have demonstrated that these requirements cannot be directly transferred to nano-enabled elicitors or plant biostimulants without sector-specific adaptation, because their intended function and assessment endpoints differ from those of biocidal products. Collection and review of information on nanomaterial-based and nano-enabled plants [13,98]. The Biocidal Products Regulation is designed for products intended to control harmful organisms, whereas nano-enabled agricultural inputs may be claimed to improve nutrient-use efficiency, abiotic stress tolerance, crop quality, plant growth, rhizosphere function, or specialized-metabolite accumulation. In these cases, the nanoscale form may contribute directly to the claimed agronomic or physiological effect, not only to the hazard profile [98]. This distinction has been emphasized by studies proposing nanoparticles and nanomaterials as plant biostimulants at appropriate concentrations, while also recognizing that excessive or unsuitable exposure may lead to phytotoxicity [13]. Recent reviews have further shown that nanoparticle effects on plants differ from those of bulk materials because of particle size, surface area, surface charge, dissolution, uptake, translocation, and transformations at root, leaf, soil, or rhizosphere interfaces [100]. Comparative studies with materials such as ZnO have also shown that bulk particles, nanoparticles, and dissolved ionic forms may produce different responses during plant growth [101]. Therefore, nano-enabled elicitors and biostimulants require assessment criteria that integrate agronomic efficacy, dose–response behavior, plant uptake and translocation, transformation products, soil and rhizosphere interactions, crop or extract residues, and downstream exposure [102]. Thus, the BPR provides a useful precedent for separate nanoform assessment, labeling, and traceability, but its requirements must be adapted to agricultural claims, plant-specific endpoints, and food or pharmaceutical production-chain exposure rather than applied directly.

In the European Union, nanospecific provisions have been incorporated into some regulatory areas but remain uneven across product categories. REACH was amended through Regulation (EU) 2018/1881 to introduce information requirements for nanoforms of substances, whereas biocidal products containing nanomaterials are subject to specific labeling and assessment provisions [103]. By contrast, fertilizing products and plant protection products appear less explicit in their treatment of nanoforms, even though nano-enabled delivery systems are increasingly discussed for fertilizers, biostimulants, elicitors, and pesticides [98]. This creates a regulatory asymmetry: nanoformulated products may be assessed differently depending on whether they are positioned as biocidal, plant protection, fertilizing, biostimulant-like, or bioinput-related products, even when similar nanoscale properties influence exposure and risk.

Scientific evidence also indicates that nanoformulation may alter biological outcomes in ways that cannot be inferred from the bulk material or the free active ingredient alone. Nanoparticle-specific transformations can determine whether nanomaterials generate beneficial, toxicity-relieving, toxic, or physiology-disturbing effects in plants, depending on dissolution, aggregation, surface modification, redox behavior, interaction with organic matter, and transformation within plant or soil matrices [41]. This is directly relevant for encapsulated elicitors because their effectiveness and safety may depend not only on the elicitor molecule, but also on carrier degradation, release kinetics, surface reactivity, mobility, and persistence under realistic use conditions.

Experimental work with nano-enabled pesticide formulations reinforces this formulation concern. For example, toxicity assessment of tebuconazole nanoformulations showed that the nanoformulated and commercial formulations may produce different toxicity patterns compared with the active substance alone, partly because formulation affects freely dissolved concentrations and exposure of non-target organisms [40]. Similarly, studies on the release and stability of tebuconazole nanoformulations in different aquatic media have shown that release and stability depend on the medium and can influence organism exposure during ecotoxicological testing [104]. Although these examples refer to nanopesticides, these phenomena show that formulation can modify exposure duration, bioavailability, target specificity, and non-target effects.

Several studies have emphasized that environmental risk assessment for nanomaterials released into agricultural systems remains underdeveloped, particularly for terrestrial matrices. Nanomaterials may interact with soil particles, organic matter, microorganisms, plant roots, and water flows, making their environmental concentrations, transformations, and biological effects difficult to predict [105]. This concern becomes especially important for nano-enabled elicitors applied to soil, seed, or foliage, because controlled-release properties may prolong the exposure of non-target organisms even when the total active ingredient dose is lower than in conventional formulations.

The Dutch National Institute for Public Health and the Environment has noted that nanopesticides may show higher bioavailability and may expose non-target organisms such as insects, worms, and soil organisms to active substances for longer periods due to controlled or slow release. It also emphasized that nanospecific properties should be included in the safety assessment for both nanosized active substances and nano-encapsulated active substances [106]. For nano-enabled elicitors, this reinforces the need to evaluate not only the active compound but also the formulation architecture and its exposure behavior.

Chitosan again illustrates why nano-enabled elicitors should not be evaluated only as “natural,” “biodegradable,” or “low-risk” materials. As a bulk polymer, chitosan can stimulate plant growth, induce defense responses, and modulate tolerance to biotic and abiotic stress [107]. However, its effects are often dose-, tissue-, species-, and system-dependent, and higher concentrations may inhibit root or shoot development or reinforce growth–defense trade-offs. When transformed into nanoparticles, chitosan may improve solubility, delivery, and functionality, but it may also modify uptake, persistence, and interaction with other encapsulated agents [91]. Therefore, the regulatory relevance of chitosan-based nanoformulations depends on the complete material profile, including polymer characteristics, molecular weight, particle size distribution, surface charge, encapsulated cargo, application route, and claimed function [108].

Several regulatory proposals have been made to address these uncertainties. One of the most influential frameworks was proposed for the regulatory evaluation of nanopesticides, arguing that nanoformulations should be compared with conventional active ingredients and assessed in terms of characterization, environmental fate, exposure, biotic uptake, ecotoxicity, and risk in aquatic and terrestrial ecosystems [85]. Subsequent work on ecological risk assessment of nano-enabled pesticides further emphasized that nano-enabled products require problem formulation, exposure assessment, effects assessment, and risk characterization adapted to their specific formulation and use pattern [109]. These principles are applicable to nano-enabled elicitors because their risk profile may differ from that of the free elicitor, particularly when the carrier modifies release, transport, or persistence.

More recent policy-oriented approaches have moved toward Safe- and Sustainable-by-Design strategies. Mech et al. argued that smart nanomaterials illustrate the broader industrial and regulatory challenges of designing safer and more sustainable advanced materials, emphasizing the need for agreed terminology, criteria, assessment tools, incentives, and regulatory preparedness [110]. For nano-enabled elicitors, this implies that regulatory thinking should begin during formulation design rather than only after product development. Parameters such as particle size distribution, release kinetics, degradation products, carrier persistence, soil mobility, worker exposure, non-target effects, and downstream residue removal should be considered early in the design process.

The 2024 EUON/ECHA report made several recommendations that are directly relevant. These include updating legislation to incorporate a nano-agrochemical definition and nanospecific considerations, establishing standardized use instructions, creating an EU-level nano-agrochemicals database, implementing a notification system for manufacturers, and requiring systematic literature reviews as well as mandatory toxicity and ecotoxicity testing to support efficacy claims and regulatory evaluation [98]. These recommendations suggest a movement from general awareness toward traceability, registration, nanospecific testing, and post-market transparency.

In the United States, the regulatory path is different but also illustrates several forms of nanospecific oversight. Under the Toxic Substances Control Act (TSCA), EPA has reviewed nanoscale materials and has used consent orders and Significant New Use Rules to limit manufacture or use under specified exposure-control conditions. These controls may include restrictions on permitted uses, personal protective equipment, engineering controls, environmental release limitations, and testing requirements to generate health and environmental effects data [111]. Reportable information for nanoscale materials includes chemical identity, production volume, manufacturing and processing methods, uses, exposure and release information, and available health and safety data [112].

For pesticide-related products, EPA has also considered nanoscale materials under the Federal Insecticide, Fungicide, and Rodenticide Act. In its Federal Register notice on pesticide products containing nanoscale materials, EPA proposed obtaining information on nanoscale materials in registered pesticide products and evaluating, on a case-by-case basis, whether a nanoscale active or inert ingredient should be considered a “new” ingredient for FIFRA and PRIA purposes, even when the same substance in non-nanoscale form is already registered [113].

More recent EPA technical work has moved toward nanospecific determination frameworks. In 2021, EPA’s Office of Pesticide Programs and Office of Research and Development described a nano-determination framework for pesticide products containing active ingredients based on metals, metal oxides, silica, or their combinations. This framework considers whether a product contains particles below one micron, whether the active ingredient can leach from the matrix, and whether it dissolves in water [114]. Although this framework does not explicitly cover all possible organic nanocarriers or elicitor-loaded systems, it shows that practical criteria are being developed to distinguish nano-enabled products from conventional formulations.

At the broader federal level, the United States also updated its National Nanotechnology Initiative Environmental, Health and Safety Research Strategy in 2024. This strategy is not a binding regulation, but it identifies federal priorities for responsible nanotechnology development, including measurement infrastructure, exposure assessment, environmental fate, human health, risk assessment, risk management, informatics, and modelling [115]. For nano-enabled elicitors, this suggests that future regulatory development will likely depend not only on product classification, but also on improved methods for characterizing nanomaterials in realistic matrices.

In Mexico, current discussions are more closely related to the broader governance of nanomaterials than to nano-enabled elicitors as a specific agricultural category. Mexico has developed NMX standards related to nanomaterials, including NMX-R-16197-SCFI-2019 on toxicological screening methods for manufactured nanomaterials, which is listed as active and entered into force in 2020 [116]. Mexico also has an NMX guide for labeling manufactured nano-objects and products containing manufactured nano-objects, although NMX standards are voluntary instruments rather than binding NOM regulations [117]. This distinction is relevant for nano-enabled agricultural inputs because voluntary standards may support characterization, labeling, or preliminary risk evaluation, but they do not necessarily ensure mandatory registration, exposure assessment, market surveillance, or nanospecific evaluation of field-applied products.

Overall, nano-enabled and encapsulated elicitors expose an emerging regulatory tension. On one hand, they may reduce active ingredient doses, improve delivery efficiency, increase elicitor stability, enhance metabolite induction, and support more sustainable plant-cell or agricultural production systems. On the other hand, their nanoscale properties may modify uptake, persistence, bioavailability, transformation, residue potential, and exposure of non-target organisms. Therefore, it is insufficient to classify these systems only by the traditional category of the active molecule or by a general biostimulant, fertilizer, plant protection, or bioinput claim. A more adequate approach should combine claim-based classification with nanospecific characterization, formulation-level assessment, exposure modelling, traceability, and lifecycle-oriented evidence.

This formulation-centered perspective provides the basis for identifying the broader critical gaps in current regulations. As advanced materials move from single active ingredients toward multifunctional systems that combine elicitors, polymers, nanoparticles, microbial metabolites, nutrients, hormones, natural extracts, and controlled-release matrices, existing regulatory categories may not be sufficient to capture their combined biological and physicochemical behavior.

## 7. Critical Gaps in Current Regulation

The discussion of nano-enabled and encapsulated elicitors illustrates a broader regulatory problem: current frameworks are not always prepared to evaluate multifunctional bioactive inputs whose behavior depends simultaneously on biological function, formulation architecture, exposure context, and claim. This challenge is not restricted to nanometric materials. However, this challenge is not restricted to nanometric materials. It also applies to conventional, micrometric, biological, and hybrid formulations whose effects may simultaneously involve nutrient-related responses, stress modulation, defense activation, growth regulation, and enhancement of specialized metabolism. Therefore, the critical regulatory gap is not only the absence of nanospecific provisions, but also the limited capacity of current frameworks to classify multifunctional bioactive inputs whose biological effects do not correspond to a single legal category.

One of the most evident gaps is the persistent mismatch between biological function and legal classification. Plant biostimulants are increasingly defined through claim-based frameworks, especially in the European Union, where Regulation (EU) 2019/1009 [51]. This represents a significant regulatory advance because it provides a clearer boundary between fertilizing products and plant protection products. However, the same boundary can be insufficient for elicitors that activate interconnected responses. In such cases, the same material may support a biostimulant claim under one formulation or use scenario, while approaching plant protection, plant growth regulation, or process-aid functions under another. This limitation reflects a broader difficulty already identified in the biostimulant literature: biological mechanisms often overlap, whereas legal categories require discrete and claim-specific classification [49].

A related gap concerns the substantiation of efficacy claims. The 2024 EN 17700 series represent an important step toward standardizing how plant biostimulant claims should be demonstrated. EN 17700-1:2024 establishes general principles for justifying product claims [63], whereas EN 17700-3:2024 provides guidance for claims related to abiotic stress tolerance [64]. Nevertheless, many regulatory systems outside the EU remain less harmonized, and even where claim-substantiation standards exist, they may not fully address the experimental complexity of elicitors. Therefore, evaluation based only on generalized growth, yield, or quality endpoints may overlook the specific metabolic or physiological pathways that justify the use of elicitors in plant biofactories or high-value phytochemical production. For these systems, claim substantiation may need to integrate not only agronomic endpoints but also metabolite yield, biosynthetic pathway activation, product consistency, residual elicitor levels, and downstream process compatibility [118].

Another gap is the uneven treatment of microbial and non-microbial inputs. Biofertilizers definition as microbial products that improve nutrient acquisition or nutrient availability, creates uncertainty for microbial elicitors, microbial consortia, microbial metabolites, and gene-edited microorganisms that may simultaneously improve nutrient use, modulate stress responses, induce plant defense pathways, or alter specialized metabolism. Recent reviews on microbial inoculants highlight that definitions and regulations remain inconsistent across jurisdictions, especially because microbial products may be described as biofertilizers, bioinoculants, biostimulants, biopesticides, or biological control agents depending on their mechanism and claim [37]. In Canada, for example, microbial supplements containing microorganisms as active ingredients require registration prior to importation or sale, including microbial consortia and genetically modified microorganisms [79]. However, not all jurisdictions provide an equally clear route for distinguishing microbial biofertilizers, microbial biostimulants, biocontrol products, and process inputs used in contained plant cell systems.

Nano-enabled formulations expose an additional and more specific regulatory limitation. As discussed in the previous section, nanoformulation may alter the identity of the regulatory object by changing solubility, stability, uptake, degradation, mobility, persistence, and residue profiles. However, agricultural regulations for fertilizing products and plant protection products do not always contain explicit provisions for nano-enabled inputs. The 2024 EUON/ECHA report on nanomaterial-based and nano-enabled plant protection, biocidal, and fertilizing products identified knowledge gaps related to applications, exposure, and hazards, and noted that the EU plant protection and fertilizing product frameworks still lack specific provisions for nanoforms comparable to those present in the Biocidal Products Regulation [98]. This gap is particularly relevant because the same active substance may generate different exposure and risk profiles when used as a free molecule, bulk material, encapsulated compound, or nanostructured formulation.

Consequently, the active compound cannot be evaluated independently from its delivery system. A salicylate, jasmonate, chitosan derivative, microbial metabolite, or plant-derived compound used as a free elicitor in a contained culture system is not necessarily equivalent to the same compound incorporated into a foliar nanocarrier, soil-applied composite, or slow-release formulation. Encapsulation, nanosizing, polymeric carriers, chitosan nanoparticles, and controlled-release composites may modify uptake routes, tissue distribution, release kinetics, environmental persistence, and non-target exposure [41]. Recent literature on nano-enabled agriculture has also emphasized that standardized international rules remain limited and that communication gaps between developed and developing countries hinder the development of consistent governance criteria for agricultural nanomaterials [105].

A further limitation is the lack of integrated criteria for distinguishing contained biotechnological use from open-environment applications. In plant cell biofactories, elicitors may be used transiently under controlled bioreactor conditions, and the main regulatory issues may involve process validation, residual levels, downstream purification, worker safety, genetic or metabolic stability, and final product consistency [119]. In contrast, field-applied elicitors, biostimulants, bioinputs, or nano-enabled formulations require consideration of soil persistence, water mobility, non-target organisms, crop residues, environmental transformation, and post-application exposure [120]. Investigations on bioreactor-based plant cell culture emphasize that scale-up, process control, product recovery, and reproducibility remain central challenges for bioactive compound production, whereas nano-enabled agriculture literature highlights the need for fate, exposure, and ecotoxicological assessment under realistic environmental conditions [121]. Current regulatory systems tend to classify products according to market category or claim, but they do not always differentiate sufficiently between these exposure contexts. This distinction is essential for gene-edited or metabolically optimized plant cell platforms, where the elicitor may function as a process input rather than as an agricultural product.

Post-market traceability and transparency also remain limited. For conventional biostimulants, the primary regulatory emphasis is often placed on composition, labeling, and efficacy evidence before commercialization. For nano-enabled or encapsulated inputs, however, additional questions arise regarding whether products containing nanostructures are identifiable in public databases, whether users and regulators can distinguish nanoformulated products from conventional formulations, and whether adverse effects can be traced back to formulation-specific properties. The EUON/ECHA report recommended regulatory updates, standardized use instructions, an EU-level nano-agrochemicals database, notification systems for manufacturers, and mandatory toxicity and ecotoxicity testing to support regulatory evaluation [98].

This regulatory gap is more pronounced in countries where technical standards exist but are not legally binding. In Mexico, NMX standards related to nanomaterial terminology, labeling, occupational risk management, risk evaluation, and toxicological screening have been developed; however, these instruments remain voluntary rather than binding NOM-based requirements. Mexico has NMX-R-16197-SCFI-2019 on toxicological screening methods for manufactured nanomaterials [116] and an NMX guide for labeling manufactured nano-objects and products containing manufactured nano-objects [117]. This situation is relevant for nano-enabled agricultural inputs because voluntary standards may support characterization, labeling, or preliminary risk evaluation, but they do not necessarily ensure mandatory registration, exposure assessment, market surveillance.

The current development of new materials makes these gaps increasingly significant. Such products may be designed to improve nutrient efficiency, mitigate abiotic stress, induce defense priming or enhance delivery precision [89]. However, regulatory systems that rely on a single declared claim may fail to capture the combined biological and physicochemical behavior of these formulations. This creates a risk of underclassification, in which products are assessed under a less demanding category despite having broader physiological, ecological, or exposure implications.

These gaps show that current regulation is evolving but remain unevenly prepared for the convergence of elicitation, gene editing, microbial technologies, biostimulant claims, and nano-enabled delivery systems. The central challenge is not only to define new categories, but to develop regulatory approaches capable of integrating material identity, biological function, formulation architecture, exposure context, claim substantiation, traceability, and lifecycle behavior.

## 8. Conclusions and Future Perspectives

The regulation of elicitors, plant biostimulants, biofertilizers, bioinputs, and nano-enabled agricultural inputs is entering a period of accelerated transition. This transition reflects the rapid development of plant-based production technologies and advanced formulations but also exposes the limitations of regulatory systems that were originally designed around more clearly separated categories such as fertilizers, pesticides, plant nutrients, or microbial inoculants. Although important progress has been made through the formal recognition of plant biostimulants, the development of claim-substantiation standards, the emergence of bioinput frameworks, and increasing attention to nanoforms, current approaches remain uneven across jurisdictions and are still insufficient to fully address the multifunctional nature of bioactive materials used in plant systems.

From this investigation, it is identified that the regulatory identity of these materials cannot be determined only by their origin, chemical nature, or biological mechanism. Instead, classification depends on the interaction between material identity, formulation, intended use, exposure scenario, and declared claim. This is particularly relevant for elicitors such as jasmonates, salicylates, chitosan derivatives, microbial metabolites, cyclodextrin complexes, and nano-enabled systems, which may simultaneously influence nutrient-related processes, abiotic stress tolerance, defense priming, growth regulation, and specialized metabolism. Therefore, a molecule or formulation that is scientifically described as an elicitor may be interpreted differently in regulatory practice depending on whether it is used as a contained process input, a plant biostimulant-like product, a plant protection-related substance, a microbial product, or a broader bioinput.

The global regulatory landscape reveals both convergence and fragmentation. Most regions are moving toward clearer product identity, improved labeling, efficacy substantiation, safety information, and, in some cases, pre-market registration. However, the regulatory entry point differs substantially among jurisdictions. Some frameworks classify these materials as fertilizing products or supplements, others as bioinputs, plant nutrients, microbial products, plant regulators, or plant protection-related substances. As a result, comparable products may be subject to different levels of regulatory scrutiny depending on where they are marketed, how they are formulated, and which claim is attached to their use. This variability can create uncertainty for innovation, particularly when plant cell biofactories and agricultural applications use similar compounds under very different exposure conditions.

Future regulatory development should therefore move toward more adaptive, evidence-based, and context-sensitive frameworks. Several priorities can be identified: clearer differentiation between scientific function and legal claim; harmonized terminology for elicitors, biostimulants, biofertilizers, bioinputs, and nano-enabled formulations; stronger criteria for efficacy substantiation; nanospecific characterization and risk assessment; improved traceability and post-market transparency; and explicit distinction between contained biotechnological use and open-environment application. These elements would help reduce under classification risks and support more consistent evaluation of multifunctional materials whose effects cannot be captured by a single regulatory category. Elicitation, metabolic engineering, and nano-enabled delivery systems should not be evaluated only as productivity tools, but also as variables that may influence product identity, reproducibility, safety, containment, and downstream classification.

Finally, these materials offer significant opportunities for sustainable agriculture and for the controlled production of pharmaceutically important compounds in plant systems. However, their responsible development will require regulatory systems capable of keeping pace with material innovation. Strengthening harmonization, claim substantiation, formulation-level assessment, traceability, and nanospecific evaluation will be essential to support innovation while ensuring safety, reproducibility, environmental protection, and regulatory clarity. This transition from molecule-centered classification toward formulation- and regulation will be critical for the future development of advanced bioactive inputs.

## Figures and Tables

**Table 1:** Comparative overview of selected regulatory frameworks relevant to elicitors, plant biostimulants, biofertilizers, bioinputs, and nano-enabled inputs.

Jurisdiction	Regulatory Approach	Distinction of Elicitor or Biostimulant-like Products
European Union	Harmonized fertilising product framework; plant protection regulated separately.	Classification depends mainly on claim: biostimulant vs. plant protection.
United States	Fragmented federal and state-level approach involving fertilizers, soil amendments, beneficial substances, plant regulators, and pesticides.	No single harmonized federal biostimulant category exists. The regulatory route depends mainly on label claims and whether the product implies plant regulation or pesticidal activity.
Canada	Fertilizers and supplements framework, with pre-market registration for several products and specific attention to microbial supplements.	Market representation is decisive. Products may be regulated as fertilizers or supplements, and microbial products generally require clearer identity, safety, and registration evidence.
Brazil	Broad bioinput framework under Law No. 15.070/2024.	The bioinput concept is broader and may include products, processes, and technologies of biological or biotechnological origin.
India	Fertilizer Control Order framework, including biofertilizers and biostimulants.	Biofertilizers are linked mainly to microbial nutrient functions, while biostimulants require specific authorization within the fertilizer control system.
China	Fertilizer registration and microbial fertilizer standards, without a separate EU-like biostimulant category.	Products are generally evaluated through fertilizer- or pesticide-related routes depending on composition, efficacy evidence, and claims.
Mexico	Pesticide and plant nutrient framework; no harmonized plant biostimulant or bioinput category.	Elicitor-like products may be difficult to classify when their main claim is stress modulation, metabolic activation, or quality enhancement rather than direct nutrient supply or pest control.

## Data Availability

No new data were created or analyzed in this study. Data sharing is not applicable to this article.

## Abbreviations

The following abbreviations are used in this manuscript:

pH	Potential of hydrogen
CRISPR/Cas	Clustered regularly interspaced short palindromic repeats/CRISPR-associated systems
RNA	Ribonucleic acid
EU	European Union
EC	European Community
CAS	Chemical Abstracts Service
FIFRA	Federal Insecticide, Fungicide, and Rodenticide Act
EN	European Standard
S.O.	Statutory Order
USDA	United States Department of Agriculture
EPA	United States Environmental Protection Agency
NOM	Norma Oficial Mexicana
CE	Conformité Européenne/European conformity
CFIA	Canadian Food Inspection Agency
MARA	Ministry of Agriculture and Rural Affairs
NY/T	Recommended agricultural industry standard of China
COFEPRIS	Comisión Federal para la Protección contra Riesgos Sanitarios
REACH	Registration, Evaluation, Authorization and Restriction of Chemicals
EUON	EU Observatory for Nanomaterials
ECHA	European Chemicals Agency
TSCA	Toxic Substances Control Act
PRIA	Pesticide Registration Improvement Act
NMX	Norma Mexicana
SCFI	Secretaría de Comercio y Fomento Industrial
